# A systematic approach to understand the mechanism of action of the bisthiazolium compound T4 on the human malaria parasite, *Plasmodium falciparum*

**DOI:** 10.1186/1471-2164-9-513

**Published:** 2008-10-30

**Authors:** Karine G Le Roch, Jeffrey R Johnson, Hugues Ahiboh, Duk-Won D Chung, Jacques Prudhomme, David Plouffe, Kerstin Henson, Yingyao Zhou, William Witola, John R Yates, Choukri Ben Mamoun, Elizabeth A Winzeler, Henri Vial

**Affiliations:** 1 Department of Cell Biology and Neuroscience, University of California, Riverside, 900 University Avenue, Riverside, CA, 92521 USA; 2 Department of Cell Biology ICND202, the Scripps Research Institute, 10550 North Torrey Pines Rd, La Jolla, CA, 92037, USA; 3 Dynamique des Interactions Membranaires Normales et Pathologiques, CNRS UMR 5235, Université Montpellier II, Place Eugène Bataillon, 34095 Montpellier Cedex 05, France; 4 Genomics Institute of the Novartis Research Foundation, 10675 John Jay Hopkins Drive, San Diego, CA, 92121, USA; 5 Department of Genetics and Developmental Biology, University of Connecticut Health Center, 263 Farmington Avenue, Farmington, CT, 06030-3301, USA

## Abstract

**Background:**

In recent years, a major increase in the occurrence of drug resistant falciparum malaria has been reported. Choline analogs, such as the bisthiazolium T4, represent a novel class of compounds with strong potency against drug sensitive and resistant *P. falciparum *clones. Although T4 and its analogs are presumed to target the parasite's lipid metabolism, their exact mechanism of action remains unknown. Here we have employed transcriptome and proteome profiling analyses to characterize the global response of *P. falciparum *to T4 during the intraerythrocytic cycle of this parasite.

**Results:**

No significant transcriptional changes were detected immediately after addition of T4 despite the drug's effect on the parasite metabolism. Using the Ontology-based Pattern Identification (OPI) algorithm with an increased T4 incubation time, we demonstrated cell cycle arrest and a general induction of genes involved in gametocytogenesis. Proteomic analysis revealed a significant decrease in the level of the choline/ethanolamine-phosphotransferase (PfCEPT), a key enzyme involved in the final step of synthesis of phosphatidylcholine (PC). This effect was further supported by metabolic studies, which showed a major alteration in the synthesis of PC from choline and ethanolamine by the compound.

**Conclusion:**

Our studies demonstrate that the bisthiazolium compound T4 inhibits the pathways of synthesis of phosphatidylcholine from choline and ethanolamine in *P. falciparum*, and provide evidence for post-transcriptional regulations of parasite metabolism in response to external stimuli.

## Introduction

**Figure 1:**
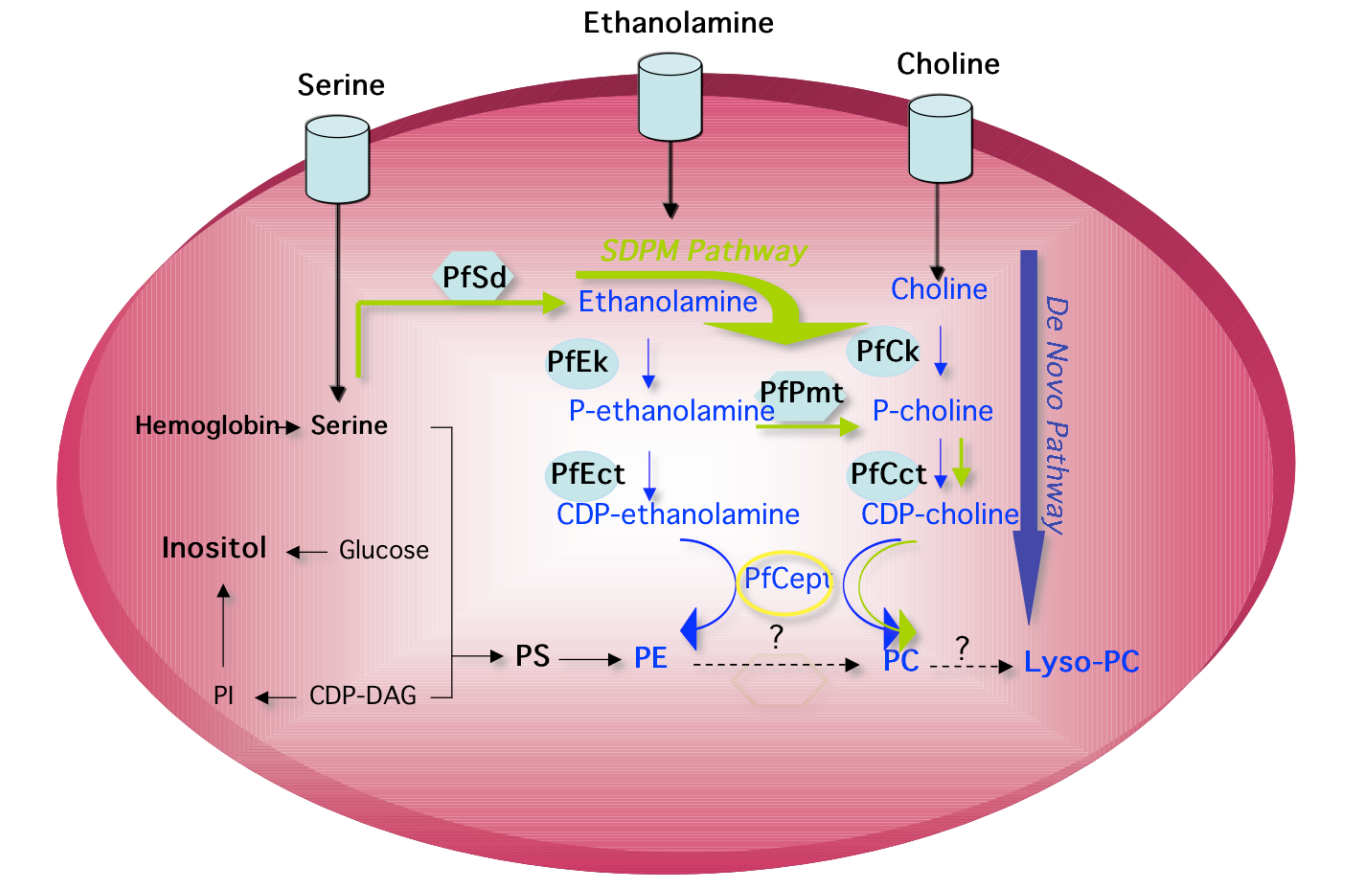
**Phosphatidylcholine biosynthesis pathways in *P. falciparum***. The figure includes relevant steps presented in the results and discussion. Enzyme and lipid abbreviations described in the figures are as followed: **PC**: Phosphatidylcholine; **PE**: Phosphatidylethanolamine; **PS**: phosphatidylserine; **PI**: Phosphatidylinositol; DAG: Diacylglycerol; **SDPM pathway**: Serine Decarboxylation-Phospho-ethanolamine Methylation; **PfSD**: Serine-decarboxylase; **PfEK**: Ethanolamine kinase; **PfPMT**: phosphoethanolamine N-methyltransferase; **PfCK**: Choline kinase; **PfCCT**: CTP-phosphocholine cytidylyltransferase; **PfCEPT**: CDP choline-choline/ethanolamine phosphotransferase protein.

Malaria remains a major public health problem in many parts of the world. Emergence and spread of resistance to widely accessible and inexpensive antimalarials make the development of novel therapeutic approaches an urgent task [1,2]. *Plasmodium falciparum *causes the most severe form of the disease and has a devastating effect on the lives of millions. Intensive asexual multiplication of the parasite occurs in human erythrocytes and is responsible for the symptoms associated with human malaria. During its 48-hour intraerythrocytic life cycle, the parasite undergoes major morphological and metabolic changes followed by nuclear division, cytokinesis and merogony to generate up to 36 new daughter parasites. Accordingly, the parasite produces and transports large amounts of lipid precursors and actively uses them to synthesize the membrane components needed during its rapid development and multiplication. Phosphatidylcholine (PC) is the major phospholipid in *P. falciparum *membranes and its content increases by approximately six-fold during the parasite's intraerythrocytic life cycle [3,4]. Because of its essential function in parasite development, multiplication and survival, its synthesis has long been viewed as an ideal target for the development of new antimalarial drugs [5]. Cell biological studies revealed that the synthesis of this phospholipid occurs via two major metabolic pathways: the *de novo *choline pathway and the serine decarboxylation-phospho-ethanolamine methylation (SDPM) pathway (Figure 1). The *de novo *choline pathway is initiated by the transport of choline from host plasma followed by three enzymatic reactions that lead to the incorporation of this precursor into PC. The SDPM pathway initiates from serine either transported from the host plasma or present within infected erythrocytes as a result of active degradation of the erythrocyte proteins by parasite specific proteases [6]. Serine is then subjected to five enzymatic reactions, two of which, serine decarboxylation and phosphoethanolamine transmethylation (phosphoethanolamine N-methyltransferase, PfPMT), are plant-like, while the last two steps are shared with the CDP-choline pathway (Figure 1) [6-8]. Because of the predicted essential role of phospholipids in the parasite's intraerythrocytic development and multiplication, several efforts have been made to synthesize lipid-based inhibitors as an alternative arsenal to fight malaria. Bisquaternary ammonium salts that mimic the structure of the phospholipid precursor choline have been shown to target membrane biogenesis in *P. falciparum *by blocking the biosynthesis of PC. The first generation of these compounds consisted of mono- and bis-quaternary ammonium salts [9,10], and their lead compound G25 efficiently inhibited the growth of drug-resistant strains of *P. falciparum in vitro*, [11], and eliminated *Plasmodium *infection without recrudescence in rodent [12] and primate models [13] at very low doses. Further structural optimization of this first generation of compounds produced the bis-thiazolium compounds T3 and T4, which, at very low doses, exerted a very rapid cytotoxic effect against malaria parasites *in vitro*. These compounds cleared malaria infection in murine and primate models with no recrudescence [14]. A clinical candidate, T3/SAR97276, has recently been advanced to clinical development in humans for treatment of severe malaria (Vial, unpublished data). To elucidate the mode of action of this promising class of compounds, we have analyzed the global effect of T4 on the parasite's transcriptome and proteome during the *P. falciparum *intraerythrocytic life cycle. No significant transcriptional changes were observed in the genes encoding those enzymes involved in PC biosynthesis. However, using a proteomic approach (MS/MS) we detected a down regulation of the choline/ethanolamine-phosphotransferase protein (MAL6P1.145, PfCEPT). Metabolic studies further confirmed the specific action of the compound on the synthesis of PC.

## Methods

### Parasite cultures, treatments and RNA extraction

*P. falciparum *parasites (3D7 strain) were cultured in human O^+ ^erythrocytes as previously described [15]. When necessary, cultures were synchronized (twice within a 6 hour interval) using sorbitol [16]. T4 was added 48 hours after the first synchronization treatment. Parasites incubated in complete medium at 8% parasitaemia and 2.5 – 3% haematocrit were treated with varying concentrations of T4. The drug was added to 25 ml cultures at 40 nM or 125 nM and the parasites were further incubated for 5, 24, 30 and 36 hours, and harvested. Total RNA was prepared as previously described [17]. Parasite synchrony and morphology were monitored by microscopic analysis of Giemsa-stained blood smears.

### Microarray analyses

The custom high-density oligonucleotide array used in this study was based on the *P. falciparum *sequence data released in July 2001 [17]. The array includes 260,596 25-mer probes corresponding to 5159 *P. falciparum *gene sequences. Probes were synthesized by Affymetrix, Inc. (Santa Clara, CA) using photosensitive oligonucleotide chemistry and photolithography. Microarray analysis was performed as previously described [17]. Briefly, eight micrograms of total RNA was used for cDNA synthesis. An oligo-dT primer containing a phage T7 promoter at its 5' end was used to prime the cDNA synthesis reaction. A second strand of cDNA was synthesized and used as a template for *in vitro *transcription in the presence of biotinylated ribonucleotide following the manufacturer instructions (Enzo). RNA hybridizations were carried out with 15 μg of fragmented cRNA at 45°C for 16 hour. The hybridization solution was removed and the arrays were stained and washed following Affymetrix protocols. Arrays were scanned with an emission wavelength of 560 nm at 3 μm resolution using a confocal scanner (Affymetrix), and the signal intensity for each sequence feature on the array was determined using Microarray Suite 5 (Affymetrix).

### Semi quantitative RT-PCR

Total RNA was extracted from synchronized untreated and treated parasites cultures after 24, 30 and 36 hours incubations with T4. RT-PCR was performed as previously described [18]. Briefly, the RNA was first incubated with Dnase I (Ambion) at 37°C for 30 min, cleaned using RNAeasy kit (Qiagen), and quantified. Double strand cDNA was synthesized using Invitogen superscript RNA amplification kit. 15 ng of cDNA were loaded into each RT-PCR reaction using the Gotaq polymerase (Promega). PCR reactions consisted of 20–35 cycles of amplification using primers chosen from the 5' coding region of each selected gene. The following primers were used: PF14_0708: forward primer 5'- cactgatgatgatattcctc, reverse primer 3' – attgtgctttaagatgatctc; PF11_0509: forward primer 5' – caagggagttcaatccact, reverse primer 3'- tcttcttgtgcatcttttgc; Mal7P1.100: forward primer 5' – gaaggggatacacttagaat, reverse primer 3' – gttctcgtattaacatgctc; Mal13P1.129: forward primer 5'- gtggttgtgatattcttctc, reverse primer 3'- atataccctacacaacgaag; Mal7P1.6: forward primer 5'- cagatgaaacaaatcctactt, reverse primer 3'- ctaatcattggatggtcttc; Mal13p1.86 (PF13_0092): forward primer 5'- gagaatcaagatgtagatcc, reverse primer 3'- tctgtagttgatacaccttc; PFF1375c (Mal6P1.145): forward primer 5'- atgtgtcttaagcgtaacca reverse primer 3'- gtatggttgtacaataagcc. Gel images were quantified for relative band intensity using the NIH ImageJ software.

### OPI algorithm

The Ontology-based Pattern Identification (OPI) algorithm was introduced by Zhou et al. and has been previously applied to the full life cycle transcriptome data set [19]. Briefly we applied the OPI analysis to six T4 transcription hybridizations along with previous 38 life cycle hybridizations. For each gene ontology group, OPI identifies a list of genes that not only share a similar expression profile, but also show functional enrichment in the target GO category. A total of 290 clusters were retained after 100 permutation runs (with estimated P ≤ 0.05). The representative expression patterns of each OPI clusters were hierarchically clustered as previously described [20]. This enabled us to visualize drug-related global expression changes side-by-side with the transcriptome expression variation in *P. falciparum *life cycle.

### Protein preparation and MudPIT

Equal amounts of untreated or T4-treated parasites were analyzed using MudPIT as previously described [21,22]. Briefly, parasite pellets were lysed by osmotic shock in 10 mM Tris-HCl (pH 8.5) and incubated in ice for one hour. After centrifugation at 18,000 g for 30 min, supernatants were saved and the pellet was solubilized in 0.1 M sodium carbonate (pH 11.5) and incubated on ice for an hour. Solubilized proteins were extracted after centrifugation and the pellet was resuspended one more time in 0.1 M sodium carbonate (pH 11.5). The three protein fractions were denatured in 8 M urea, reduced in 5 mM Tris (2-carboxyethyl)phosphine hydrochloride (TCEP, Roche); alkylated by 20 mM iodoacetamide (IAM), and digested with proteinase K (Roche) for 4 hours at 37°C, in 0.1 M Sodium Carbonate (pH 11.5) as described in Wu et al. 2003 [23]. Peptide mixture was analyzed as previously described [24].

### Targeted MS/MS approach

Nine proteins involved in key phospholipid biosynthesis were selected (PFL0620c; MAL13P1.214; PF11_0257; PF13_0253; PF14_0020; PF13_0092; MAL6P1.145; PFI1370c; and MAL8P1.58). Ten peptides uniquely matching these proteins were targeted for quantitation in a directed MS/MS experiment. Treated and untreated parasites were lysed with 0.1 M Na_2_CO_3_, centrifuged for 30 minutes at 4°C, and fractionated into soluble and pellet fractions. After adjusting the pH to 8.5, fractions were denatured, reduced, alkylated and digested with endoproteinase Lys-C and trypsin as described previously [21].

The chromatography method entailed a six-step MudPIT approach, and a custom MS method collecting MS/MS spectra for each of the 10 targeted ions repeatedly. To estimate the rate of false positives in the protein identification analysis, MS/MS data were searched using SEQUEST against a combined database containing *P. falciparum *and Human/Mouse/Rat amino acid sequences as well as an identical database where each protein sequence was randomized. The length and amino acid composition of each protein sequence remained the same, but the positions of amino acids were altered. Thus any hits to the scrambled protein sequences indicate false positives, and the overall rate can be determined. Protein identification parameters were adjusted to achieve a 5% false positive rate.

For the *P. falciparum *phosphoethanolamine methyltransferase, PfPMT, peptides derived from untreated and T4-treated samples eluted in the same MudPIT cycle with retention times that correlated with each other – eluting at 54 and 58 minutes for untreated and treated samples, respectively. Extracted ion chromatograms (XICs) for the y6, y8, y10, and y15 fragment ions of PfPMT peptide were generated for the cycle in which they were identified using a retention time window of two minutes (Data not shown). Each individual XIC was smoothed using adjacent averaging, and the area under each was calculated. The average ratio was 1.03 (untreated:treated) with a standard deviation of 0.08.

For PfCEPT, 45 spectra were identified as PfCEPT peptide in untreated sample and 1 spectra in T4-treated samples. The single peptide identified in the treated sample had a significantly different retention time (0.43 minutes) from those identified in the untreated sample, which eluted consistently near the end of the organic gradient (~113 minutes). An inconsistent retention time and manual inspection of the MS/MS spectrum led us to believe the peptide detected in the treated sample was a false positive. XICs were generated for the y5, y6, y13, and y14 fragment ions, which clearly indicate that this peptide elutes near 113 minutes in the untreated sample, whereas no signal was detected for any of these fragment ions in the treated sample near this retention time (Data not shown).

### T4 effects on P. falciparuml phospholipid biosynthesis

*P. falciparum*-infected erythrocytes (3D7 strain) (5–7% parasitemia, 5% hematocrit) were incubated in 150 μl of choline-free RPMI 1640 supplemented with 25 mM Hepes (pH 7.6) in the presence or absence of T4 at the indicated concentration. After 30 minutes, 50 μl of medium containing [^3^H]-choline (5 μM final concentration in 200 μl, 2 μCi/well), [^3^H]-ethanolamine (2 μM, 1 μCi/well) or [^3^H] hypoxanthine (1 μCi/well) were added. Suspensions were incubated for 4 hours at 37°C in a 5% CO_2 _incubator. Reactions were stopped at 4°C and cell suspension washed 3 times with 0.9% NaCl. Phospholipids were extracted according to a modified Folch protocol [25,26] and the organic phase was fractionated by silica gel thin-layer chromatography (TLC) with chloroform/methanol/acetic acid/0.1 M sodium borate (75/45/12/4.5) [26]. Identified phospholipids were scraped off and radioactivity was measured by scintillation counting.

### T4 effects on aqueous-soluble metabolites

*P. falciparum*-infected erythrocytes were incubated at 20 – 22% hematocrit and 5–10% parasitemia in a final volume of 200 μl of choline-free RPMI 1640 supplemented with 25 mM Hepes (pH 7.6) in the presence or absence of T4. [^3^H]-choline (20 μM) and [^3^H]-ethanolamine (2 μM) were added at time 30 min. Suspensions were incubated for 2.5 hours at 37°C in a 5% CO_2 _incubator. Water-soluble metabolites were obtained from the aqueous supernatant according to the Folch protocol and were fractionated by a silica gel TLC with methanol/water/30% NH_4_OH (70/29/1) (Retention Factor Cho:0.02; P-Cho:0.18; P-Etn:0.44; CDP-Cho and CDP-Etn:0.78). Spots were identified by comparison to co-migrating standards. All spots were scraped off and amounts of radioactivity were determined by liquid scintillation counting. Results are expressed as percents of control test without T4.

## Results

### Global transcriptional analysis of T4-treated P. falciparum

**Figure 2:**
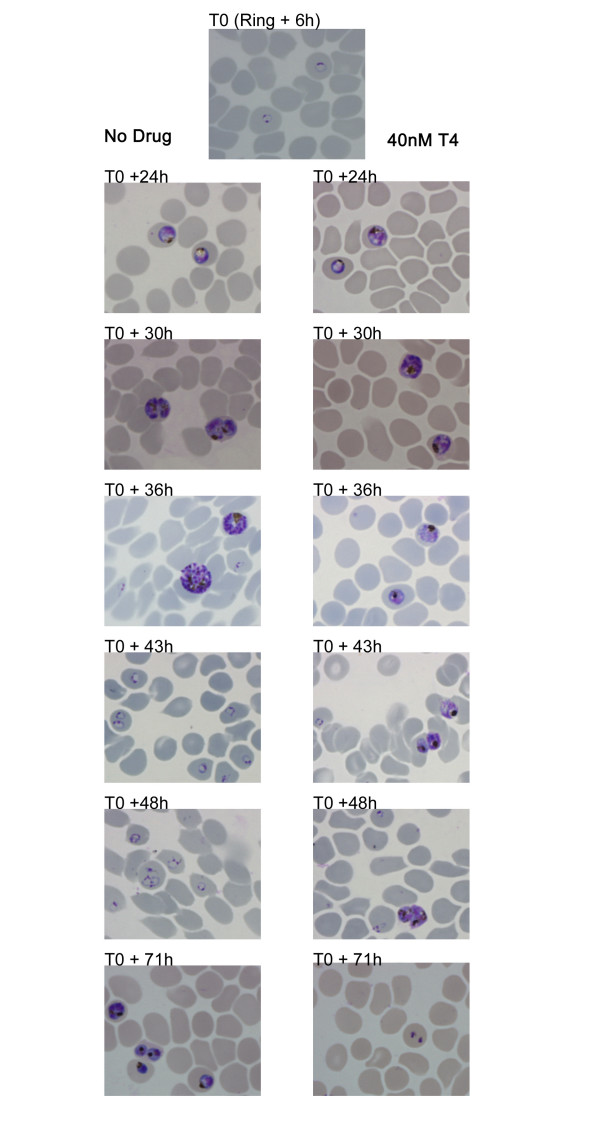
**Giemsa-stained smears of T4-treated and untreated cultures**. T4 was added at 40 nM at the early ring stage. The morphology of parasites in treated and untreated cultures is shown (early trophozoites, T0+24 h; late trophozoites, T0+30 h; schizonts, T0+36 h; early ring stage, T0+43 h; late ring stage, T0+48 h; and trophozoites, T0+71 h). Morphological changes are clearly observed at 36 hours with absence of schizont formation (T0+36 h). Between 43 and 48 hours surviving parasites are blocked at the trophozoite stages. At 71 hours parasitemia falls to negligible level.

**Figure 3:**
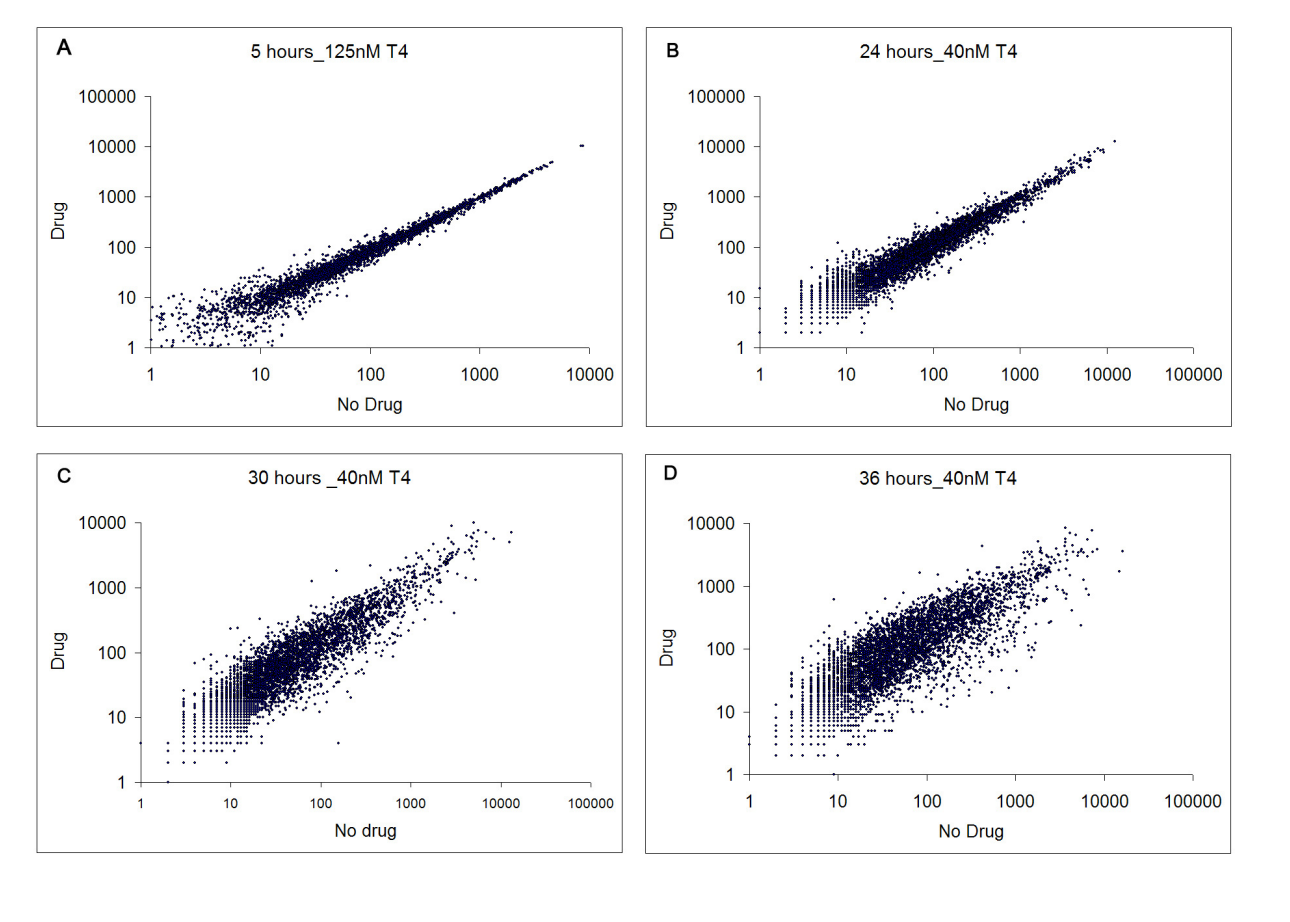
**Logarithm-transformed expression values of untreated versus treated *P. falciparum *cultures**. **A- **The logarithm-transformed expression values of untreated and treated (T4 = 125 nM) mixed population after 5 hours of incubation. **B – C – D **– The logarithm-transformed expression values of untreated and treated (T4 = 40 nM) synchronized population for 24, 30 and 36 hours of incubation respectively (drug added at the ring stage).

**Figure 4:**
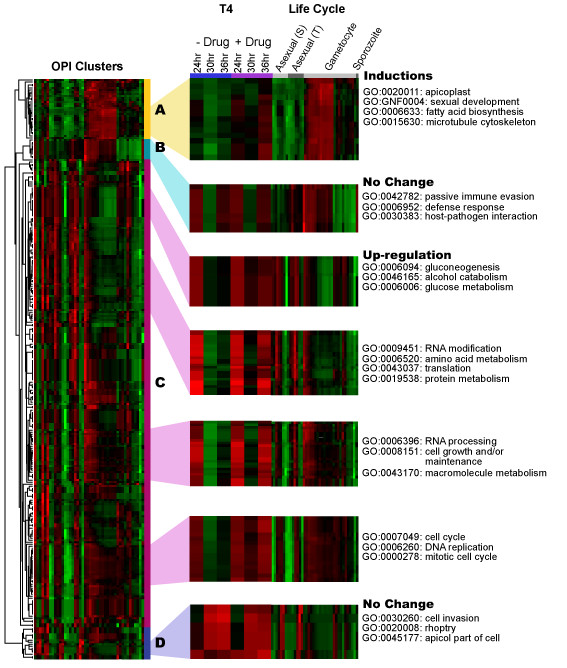
**Hierarchical clustering of all 290 OPI clusters**. Genes in each cluster share a similar expression pattern represented as a row in the heat map. The right panel is the zoom-in view of selected heat map sections with the six T4 data set further horizontally expanded for ease of visual inspection. Bright red indicates highest expression; bright green indicates lowest expression (gene-wise normalized). Asexual S, T refers to the erythrocytic cycle expression profile analysis of malaria parasite synchronized using Sorbitol chemical treatment (S) or the thermo-cycling incubation (T) describes in Le Roch et al, 2003. Gene behaviors of T4-treatment can be characterized into three categories: Pattern A shows an induction in gametocytogenesis; Pattern B and D shows no change in expression before and after T4 treatment; Pattern C shows an up regulation most likely due to DNA replication, metabolism and cell cycle progression. Some representative GO groups are shown next to the pattern.

Consistent with those results reported previously for various *P. falciparum *clones [14], T4 inhibited growth of the 3D7 strain with an IC_50 _of 1 nM. We were interested in the biological effect of this drug at early stages of the lifecycle, and over longer time periods. As such, highly synchronized cultures were harvested 6, 12, 18, 24, 30, 36, 43, 48 and 71 hours after the addition of 0 or 40 nM T4 (> IC_80 _value), to allow morphological analysis, RNA isolation and protein extraction (Figure 2). Short exposures (4 hours incubation time) with 40 nM T4 have been shown to inhibit growth of ring and trophozoite stages by 99% and 85%, respectively [14]. Despite the fact that short drug exposure shows a potent non-reversible cytotoxic effect [14], parasites were maintained in the presence of the drug to avoid additional culture handling, parasite stress (due to temperature changes) and loss of potential transcriptional effects after washes. Whereas no morphological differences could be detected between untreated and T4-treated parasites during the first 24 hours, significant morphological abnormalities and arrest of the cell cycle progression were observed between 30 and 71 hours following addition of 40 nM T4. In these cultures, small and dense trophozoites appeared and parasites failed to enter schizogony (Figure 2). Parasitemia of treated cultures decreased dramatically at 48 (3–4% parasitemia versus 10–12%) and 71 hours (0.5–1% parasitemia versus 10 – 12%). RNA extraction was performed on each sample in order to monitor global transcriptional profiles. RNA samples were labeled and hybridized to the custom malaria oligonucleotide array as previously described [17]. No significant differences were detected between untreated and T4-treated parasites during the first 18 hours of incubation at low drug concentration (2 nM and 20 nM (The correlation coefficient of the transcript expression values between untreated and T4-treated cultures was 0.99) – Data not shown). No significant changes after 5 hours of incubation with a high drug concentration (125 nM – Figure 3A and Additional file 2) were detected. Hybridization studies performed with parasites incubated with 40 nM of T4 for 24 hours resulted in a correlation coefficient of 0.98 with a total of 137 genes showing greater than 2-fold differential expression between untreated and T4-treated parasites (P_ANOVA _values for these genes were lower than 0.05 (Additional file 3 and Figure 3B). Of the 137 genes, 99 were known to be expressed in gametocytes, 21 were known to display strong variations in the level of expression throughout the erythrocytic life cycle and 17 showed a transcript level close to background in treated and untreated parasites. None of these genes were known or predicted to be involved in phospholipid metabolism in *P. falciparum *(Figure 1). T4 treatment for 30 and 36 hours resulted in increased transcriptional changes with 1140 and 1825 genes differentially expressed, respectively (See Additional file 3). The correlation coefficient of the expression values between untreated and treated parasites at these time points decreased significantly (r = 0.87 and 0.72 at 30 and 36 hours, respectively) (Figure 3C and 3D). However the correlation coefficients observed between transcripts at different stages of the *P. falciparum *cell cycle without treatment also changed significantly (i.e., 24 hours/30 hours, r = 0.64; 30 hours/36 hours, r = 0.95 and 24 hours/36 hours, r = 0.95). To further understand these complex transcriptional changes, data obtained from studies involving T4 treatment were compared to those previously reported for the parasite intraerythrocytic life cycle [17,19] using the ontology based pattern identification algorithm (OPI) [20]. As illustrated in Figure 4, three main categories of gene behavior emerged. The first category includes genes already known to be expressed during gametocytogenesis [19] (pattern A). These genes were defined by the gene ontology groups GNF0004 (sexual development), GO:0006633 (fatty acid biosynthesis in apicoplast) or GO:0015630 (microtubule cytoskeleton). The second category includes genes whose expression was not significantly altered following treatment with T4 (pattern B and D Figure 4). These include the following representative GO clusters: GO:0030260 (cell invasion), GO:0020008 (rhoptry), GO:0045177 (apical part of cell) and GO:0030383 (host-pathogen interaction). The third category (pattern C Figure 4) encompassed genes involved in cell cycle and metabolism such as GO:0006006 (glucose metabolism), GO:0019538 (protein metabolism), GO:00063396 (RNA processing), GO:0007049 (cell cycle) and GO:0006260 (DNA replication). The expression profile of the genes in this category in untreated parasites decreased during the trophozoite/schizont transition (30 and 36 hours), consistent with previous reports. In contrast, in treated parasites, these genes exhibited stable transcript levels at 30 and 36 hours. Importantly, although T4 and its analogs are known to interfere with the synthesis of PC, no specific effect on the expression of the genes encoding enzymes of the CDP-choline and SDPM pathways could be detected in response to the drug (Additional file 4). RT-PCR analysis performed on RNA isolated from untreated and T4-treated parasites confirmed the results of the microarray analyses (See Additional File 1). The complete cluster analysis as well as various statistical parameters can be accessed from our web page  or from the PlasmoDB database .

### Proteomic profiling

The lack of specific transcriptional regulation of the genes involved in phospholipid metabolism following T4 treatment led us to investigate the global effect of this compound on the proteome. To estimate relative changes in protein levels following treatment with T4, proteins were extracted from cultures 24 hours after the addition of T4 at a concentration of 40 nM to ring-stage parasites. Protein abundance was estimated by mass spectrometry analysis {Zybailov, 2005 #341; Le Roch, 2004 #203; Florens, 2002 #112}. Protein identification was carried out in six experiments, each consisting of three replicate analyses of treated and untreated parasites. A total of 889 proteins were identified, with 560 and 571 proteins identified from T4 treated and untreated parasites, respectively (Additional file 5). The Pearson correlation coefficient was high (r = 0.932) compared to the Pearson correlation coefficient observed between independent proteome datasets of different stages of the *P. falciparum *life cycle (i.e., ring/trophozoite, r = 0.534; trophozoite/schizont, r = 0.389 and schizont/ring, r = 0.506) [22]. The high correlation coefficient observed between untreated and T4-treated parasites correlates with the lack of observable transcriptional changes at 24 hours post drug treatment. Nevertheless, a small number of proteins showed subtle differences in their relative abundance. Proteins predicted to play a role in biological processes occurring in the food vacuole, such as plasmepsin or falcipain, were enriched amongst those proteins showing the most significant increases. Four of the seven annotated food vacuole proteins were among the top 64 of those that showed increased peptide abundance in the T4 treated sample. Conversely, with the exception of MSP1, many know invasion proteins such as rhoptry proteins (RAP1, CLAG1, and CLAG2) were found to be less abundant following treatment with T4 (P = 8.47e^-7^). By focusing our analysis on proteins significantly up-regulated in response to drug treatment, 53 proteins were found to exhibit a 3-fold or greater increase in expression level (as measured by the ratio of spectral counts) or were detected only in the drug treated samples. Of these 'over-expressed' proteins, nine were proteins from surface antigen families. Their modulation may represent an overall stress response of the parasite [28]. The glycerol-3-phosphate acyltransferase (PFL0620c, PfGAT) and the acyl carrier protein homolog (PFB0385w) were detected in the drug treated samples only. In addition, the PfPMT, a key enzyme in the SDPM pathway as well as the S-adenosylmethionine synthetase (PFI1090w, which provides the PfPMT essential cofactor S-adenosylmethionine), showed an increase in treated versus untreated spectral counts (17 versus 5 spectra counts for PfPMT and 7 versus 2 spectral counts for PFI1090w in T4 treated and untreated parasites, respectively). Using a semi quantitative MudPIT analysis, slight differences in spectral counts are not always significant and enzymes expressed at low levels are often difficult to detect. To improve our analysis and perform a quantitative proteomic analysis, we used a labeled isoleucine strategy [29]. Unfortunately, this method proved unsuccessful due to an irregular distribution of labeling efficiency for all proteins even after two cell cycles (Data not shown). This is most likely attributed to the incorporation of non-labeled isoleucine from the human serum. To gain further insight into the expression pattern of proteins involved in phospholipid metabolism in the absence or presence of T4, a targeted MS/MS approach was used. Ten peptides derived from nine phospholipid metabolic enzymes were selected (Additional file 6). PfPMT, and choline/ethanolamine-phosphotransferase (PfCEPT, MAL6P1.145; involved in the final step of synthesis of PC via the choline and SDPM pathways), were successfully detected [7]. The difference in the levels of PfPMT peptides detected in untreated (2 spectra) and T4-treated (6 spectra) parasites was not highly significant. In the case of PfCEPT, however, 45 peptides were detected in the absence of T4 compared whereas in the presence of the compound no peptide spectra could be detected. This result suggests that the expression of PfCEPT is severely reduced following treatment with T4. Using the global proteomic approach as well as the targeted MS/MS approach, no additional known or predicted phospholipid metabolic enzymes were detected. The abundance of these proteins may be below the levels that can be detected using mass spectrometry.

**Figure 5:**
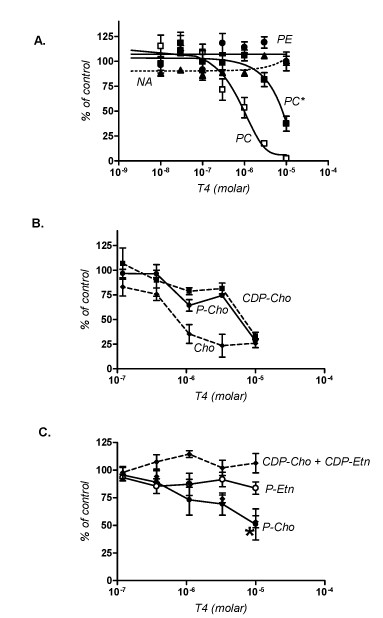
**Biochemical effect of the bisthiazoliums T4**. **A**- Effect of T4 on the biosynthesis of phospholipids and nucleic acids. *P. falciparum *trophozoite-infected erythrocytes were incubated in the presence of the indicated concentrations of T4 at time 0 and radioactive choline (5 μM), ethanolamine (2 μM) and hypoxanthine were added at time 30 min. Incorporation of the radioactive precursors into their corresponding macromolecules at time 4 h are expressed as percentage of those measured in infected erythrocytes incubated without T4 and are means of 4 experiments ± SEM. In the absence of drugs, choline incorporation into PC was 4.71 ± 0.26 nmol/10^10 ^cells/hour, and ethanolamine incorporation into phosphatidylethanolamine PE and into PC (indicated as PC*) was 14.3 ± 0.33 and 1.97 ± 0.16 nmol/10^10 ^cells/hour. Effects of T4 on choline (B) and ethanolamine (C) water-soluble metabolites. *P. falciparum*-infected erythrocytes were incubated in the presence of the indicated concentrations of T4 at time 0. Radioactive choline (20 uM) and ethanolamine (2 uM) were added at time 30 min and reactions were stopped at time 2.5 hours. Incorporation of the radioactive precursors into their corresponding metabolites is expressed as percentage of those measures in infected cells incubated without T4 and are means of 4 experiments ± SEM. In the absence of drugs, intracellular choline, phosphocholine and CDP-choline were respectively 1.1 ± 0.1, 23.7 ± 0.5 and 0.30 ± 0.01 nmol/10^10 ^cells/hour. In C, phosphorylethanolamine and phosphocholine and the sum CDP-choline + CDP ethanolamine were respectively 4.90 ± 0.25 and 1.86 ± 0.07 and 0.77 ± 0.06 nmol/10^10 ^cells/hour. Incorporation of Eth into P-ETn is not affected by T4.

To validate T4 specific effects on PC metabolism, the incorporation of radiolabeled choline (Cho) and ethanolamine (Etn) into PC and phosphatidylethanolamine (PE), and their intermediary water-soluble metabolites, phosphocholine (P-Cho), phosphoethanolamine (P-Etn), cytidine-diphospho-choline (CDP-Cho) and cytidine-diphospho-ethanolamine (CDP-Etn) was monitored in the absence or presence of the compound. As shown in Figure 5A, choline-derived PC levels were decreased by 50% in the presence of 0.8 μM T4. At this concentration, the synthesis of PC from ethanolamine was unaffected. Higher concentrations of T4, however, resulted in a decrease in PC biosynthesis from ethanolamine. On the other hand, T4 had no affect on the biosynthesis of PE or nucleic acid from ethanolamine or hypoxanthine, respectively. Together these data suggest that T4 treatment elicits a rapid and specific inhibition of the synthesis of PC via the two main biosynthetic pathways. Analysis of the water-soluble intermediary metabolites of PC biosynthesis following labelling with [^14^C]choline showed that T4 treatment caused a major decrease in the intracellular levels of radiolabeled choline and of the downstream metabolites phosphocholine and CDP-choline (Fig. 5B and 5C). This suggests that T4 may have an effect on the transport of choline into the parasite and may also inhibit the first two steps of the CDP-choline pathway catalyzed by the the choline kinase, PfCK1 and the phosphocholine citidylyltransferase, PfCCT. The inhibition of choline transport is consistent with previous studies that showed inhibition of choline transport into *P. falcparum*-infected erythrocytes and yeast by quaternary ammonium compounds [22,27]. The metabolic studies also showed that at T4 concentrations above 1 μM the incorporation of radiolabeled ethanolamine into phosphocholine was significantly decreased whereas ethanolamine phosphorylation was not affected, suggesting an inhibition of at least one enzyme downstream P-Etn i.e. PfPMT, CTP-phosphocholine cytidylyltrasnferase (PfCCT) or PfCEPT.

## Discussion

In the recent years, large-scale transcriptional profiling has been used, with varying degrees of success, to gain a better understanding of the mechanism of action of small molecules [30-35]. Whereas this approach has sometimes been challenging [36], in large part it has proven successful in bacteria and the yeast *Saccharomyces cerevisiae*. In multicellular organisms, large-scale transcriptional profiling analyzed under different stress conditions have proven more difficult due to multiple cell types or tissues as well as complex transcriptional regulations. In addition to transcriptional profiling, proteomic approaches are also gaining wide use as molecular tools to help unravel the mode of action of various compounds [37-39]. In this study, we have combined both approaches to study the response of the human malaria parasite, *P. falciparum*, to exposure to the bisthiazolium choline analog T4. Following exposure to low concentrations (2, 20 nM, (data not shown) or short incubations at 125 nM (30 minutes and 5 hours – data not shown), no specific transcriptional changes were observed, nor after a 5 hour treatment at a higher dose (T4 = 125 nM; Figure 2 and Additional File 2). Following a longer incubation with 40 nM T4 (24, 30 and 36 hours) no specific changes could be observed in the transcription of genes involved in phospholipid metabolism. However, changes due to an arrest of the cell cycle were significantly detected after 30 and 36 hours of incubation with the drug. To subtract the transcriptional changes due to cell cycle effect, we analyzed the transcription profile obtained at 24, 30 and 36 hours with previously published life cycle data set [19,22] using the OPI algorithm [20]. A transcriptional profile reminiscent of a global drug-induced stress response was detected when compared to untreated cultures (Figure 4). This profile is characterized by an apparent arrest of the cell cycle and a significant induction of genes known to be expressed in gametocytes [17,19]. This cell cycle arrest could signify a major shift of the parasite from asexual development to sexual differentiation. Although more than 5,000 infected red blood cells were analyzed, no gametocytes could be observed in the treated cultures, suggesting a specific induction of expression of genes involved in sexual differentiation and development rather than production of gametocytes in the culture following drug treatment. Gametocytogenesis can be induced by various external factors both *in vitro *and *in vivo*, most of which are conditions that cause physiological stress on the parasite, such as treatment with antimalarial drugs [40-45]. Thus, the changes in gene expression observed as a result of T4 treatment might indicate an attempt by the parasite to commit to sexual differentiation. The lack of gametocytes following treatment with T4 could, however, be due to the potent activity of quaternary ammonium compounds against this stage of the parasite's life cycle or the time taken to produce gametocytes post commitment to gametocytogenesis. However, G25, the first generation lead compound [13] was shown to prevent *P. falciparum *sexual differentiation and to prevent parasite transmission by altering gametocyte maturation, exflagellation and sporogony (H. Vial, personal communication). Using the OPI algorithm, our data showed that, once changes due to the cell cycle and stress related (gametocytogenesis) genes were subtracted, T4 treatment led to no specific or compensating changes in transcription under the experimental conditions described herein. It is possible, however, that a longer exposure to a lower drug concentration could reveal a parasite specific response. Using such a condition, Dahl et al [46] were successful in their use of transcriptome analysis to identify the effects of tetracycline on the *P. falciparum *apicoplast after more than 48 hours of treatment.

Global proteomic profiles of untreated and T4-treated parasites after 24 hours suggested that T4 treatment leads to an induction of those proteins localized to the food vacuole. As choline analogs have been shown to accumulate within the parasite digestive vacuole and possibly interact with heme [47], detection of an increased level of these enzymes potentially validate this interaction. A decrease in the levels of invasion proteins such as those localized to the rhoptry or the peripheral surface of the parasites was also detected. This could suggest a cell cycle arrest at the protein level even thought no significant transcriptional changes were detected for such proteins. To assess the effect of T4 on the expression of enzymes involved in phospholipid metabolism, changes in protein levels using a targeted MS/MS approach was applied. The proteomic data indicated a significant decrease in the abundance of PfCEPT, a protein involved in the final step of phosphatidylcholine synthesis. Nonetheless, no PfCEPT transcriptional changes could be detected at 24 hours using RT-PCR analysis (data not shown). Previous studies have shown a significant discrepancy between mRNA and protein levels, suggesting a major role for post-transcriptional events within the parasite life cycle [22,48,49]. One possible explanation for the effect of T4 on PfCEPT is that the expression of PfCEPT could be directly controlled by the amounts of CDP-choline available.

Metabolic studies showed that T4 inhibits the biosynthesis of PC but not that of PE or DNA. This finding is consistent with previous reports that showed a specific inhibition of PC biosynthesis by quaternary ammonium compounds [11,14,50-52]. Previous studies have shown that T4 accumulates to 200 to 300-fold in *Plasmodium*-infected erythrocytes within two hours [14]. This suggests that the concentrations used in the metabolic studies are within the range of the intracellular concentration of the compound, and that the pathways for synthesis of PC from both choline and ethanolamine are the targets of the compound. These biochemical analyses showed that T4 treated parasites had low levels of intracellular choline, phosphocholine and CDP-choline. This suggests that T4 inhibits choline transport and may also act by inhibiting the steps of the CDP-choline pathway catalyzed by the choline kinase PfCK and the phosphocholine cytidylytransferase, PfCCT. The inhibition of choline entry into the parasite is consistent with previous findings that showed that this class of compounds are potent inhibitors of choline transport into Plasmodium-infected erythrocytes and into the parasite [52,53]. The metabolic studies also showed that at a slightly higher concentration T4 inhibited the synthesis of phosphocholine from ethanolamine with no effect on the synthesis of phosphoethanolamine or CDP ethanolamine. This point out an additional effect of T4 that also inhibiting PC biosynthesis from ethanolamine through an action on one step downstream P-Etn, i.e. PfPMT, CTP-phosphocholine cytidylyltransferase (PfCCT) or PfCEPT. In vitro studies indicated that millimolar concentrations of the bisthiazolium are required to dose-dependently inhibit the PfCCT and PfCEPT (unpublished). Thus, the very high accumulation of the choline analogues into the blood stage of the malarial parasite [13,14], can lead to *in situe *concentration able to interfere with parasite choline uptake and PC biosynthetic enzymes. The specificity of inhibition by T4 towards enzymes of the CDP-choline pathway and eventually PfPMT whose substrates or products contain a choline moiety could thus be directly linked to its structural properties. Future structural studies aimed to characterize the interaction between T4 and these enzymes are warranted.

The proteomic data indicated that PfCEPT protein levels decreased significantly following treatment with T4. Taken together these findings suggest that PfCEPT expression or stability might be directly regulated by the availability of CDP-choline. Recent studies have provided evidence for the regulation of the expression and stability of PfPMT in response to exogenous choline [54]. It is possible that PfCEPT might also be subjected to posttranslational regulation by its substrate CDP-choline. Low amounts of this substrate due to direct inhibition of PfCCT or other earlier steps of the CDP-choline and SDPM pathways could result in the inhibition of synthesis or increased degradation of PfCEPT.

In conclusion, despite the absence of highly significant transcriptional changes within the metabolic pathways targeted by T4, this analysis validates the existence of a specific transcriptional cascade across the *P. falciparum *cell cycle [17,55]. The malaria parasite, as an obligate intracellular parasite, has evolved within a buffered intracellular environment where evolutionary forces may have induced the loss of specific transcriptional feedback responses. Proteomic and biochemical studies indicate a decreased level of PfCEPT, suggesting a specific mechanism of inhibition of PC biosynthesis. Together, these results emphasize the importance of post-transcriptional regulation in parasite drug response. The nature of such modifications has been largely unexplored in *P. falciparum*. It can involve mRNA stability, phosphorylation, as well as an increase in protein instability and degradation via the ubiquitin/proteasome pathway. Further experiments will be required to characterize the mechanisms involved in such post-transcriptional regulations.

## List of abbreviations

**MudPIT**: Multidimensional Protein Identification Technology; **OPI**: Ontology-based Pattern Identification algorithm; **PC**: Phosphatidylcholine; **SDPM pathway**: Serine Decarboxylation-Phospho-ethanolamine Methylation; **PfPMT**: phosphoethanolamine N-methyltransferase; **PfCEPT**: choline/ethanolamine-phosphotransferase protein; **Cho**: Choline; **Etn**: Ethanolamine; **PE**: Phosphatidylethanolamine; **P-Cho**: Phosphocholine; **P-Etn**: Phosphorylethanolamine; **CDP-Cho**: Cytidine-diphospho-choline; **CDP-Etn**: Cytidine-diphospho-ethanolamine; **PS**: Phosphatidylserine; **PfSD**: Serine-decarboxylase; **PfEK**: Ethanolamine kinase; **PfECT**: CTP: Phosphoethanolamine cytidylyltransferase; **PfCK**: Choline kinase; **PfCCT**: CTP:phosphocholine cytidylyltransferase; **XIC**: Extracted ion chromatography; **TLC**: Thin layer chromatography.

## Authors' contributions

KLR conceived the study and the design, carried out microarray and proteomic studies, participated in bioinformatics analyses and wrote the manuscript. JRJ carried out proteomic studies and conducted proteomic statistical analyses. HA carried out metabolic studies. DDC and WW carried out molecular studies. DP and KH carry out parasite cultures. YZ conducted statistical and bioinformatics analyses. JRY edited the manuscript. CBM helped in the design of molecular studies and edited the manuscript. EAW participated in the microarray design and edited the manuscript. HV conceived the study, the design and edited the manuscript. All authors read and approved the manuscript.

## Supplementary Material

Additional file 1 **Additional Figure 1**: **RT-PCR validation **– RT-PCR experiments were performed to validate the microarray results. *Plasmodium *RNA was extracted at 24, 30, and 36 hours after 40 nM T4 treatment of synchronized cultures. Untreated RNA was extracted in tandem as a control. cDNA was created from the RNA extractions and quantified using spectrophotometry. 15 ng of cDNA were loaded into each RT-PCR reaction and was subjected to 20–35 cycles of amplification. Semi-quantifications of RT-PCR were done using NIH's ImageJ and illustrated by a column graph. Another column graph depicting the microarray results for the respective genes is shown for comparison. Pf14_0708, Mal13P1.129 and Mal7P1.100 were selected as genes expressed in gametocytes and showing a significant induction when incubated with T4); PF11_0509 and Mal7P1.6 showed an arrest of the cell cycle progression. Mal13P1.86, a gene involved in the parasite lipidic pathway is expressed and does not show any particular transcriptional change when incubated with the drug. Click here for file

Additional file 2 **Additional table 1: Transcriptional intensities at 30 minutes and 5 hours time points for treated and untreated parasite at 125 nM of T4 on a mix parasite population.** Click here for file

Additional file 3 **Additional table 2: Transcriptional intensities at 24, 30 and 36 hours time points for treated and untreated parasite at 40 nM on synchronized parasites.** Click here for file

Additional file 4 **Additional table 3: Known and putative phospholipid and neutral lipid enzymes of P. falciparum.** Click here for file

Additional file 5 **Additional table 4: Protein abundance detected at 24 hours for treated and untreated parasite at 40 nM of T4 on synchronized parasites.** Click here for file

Additional file 6 **Additional table 5: Nine proteins selected for the Targeted MS/MS approach.** Click here for file
