# Supplementary figures and images for: A systematic approach to understand the mechanism of action of the bisthiazolium compound T4 on the human malaria parasite, Plasmodium falciparum
Source: BMC Genomics. 2008 Oct 30;9:513. doi: 10.1186/1471-2164-9-513 (PMC2596145; doi:10.1186/1471-2164-9-513)

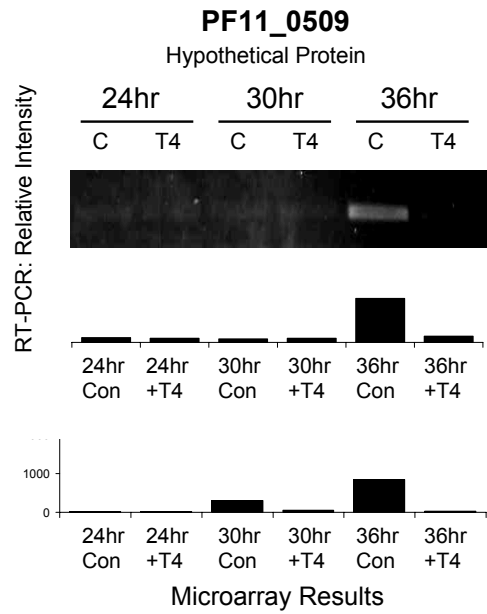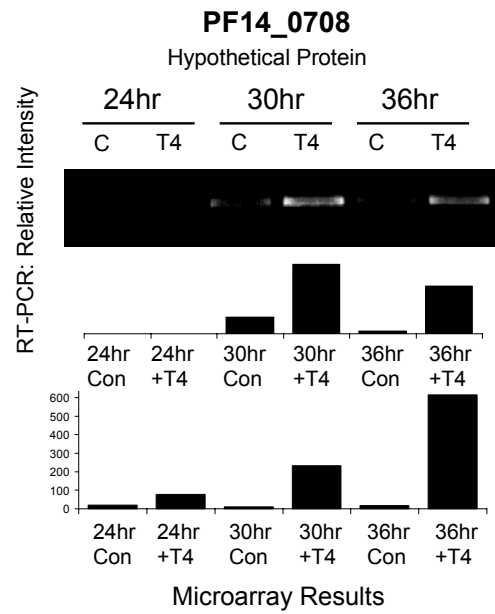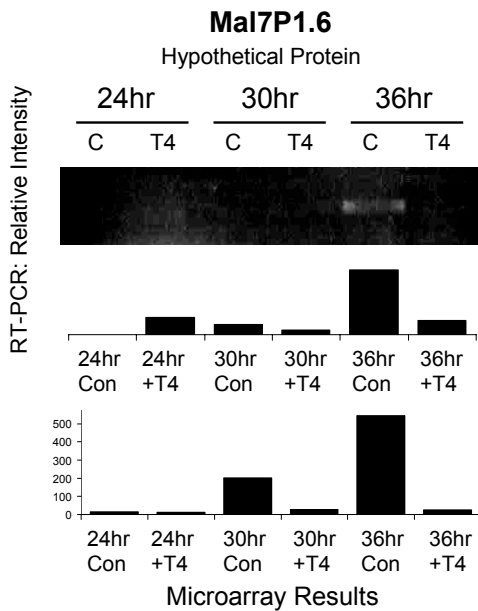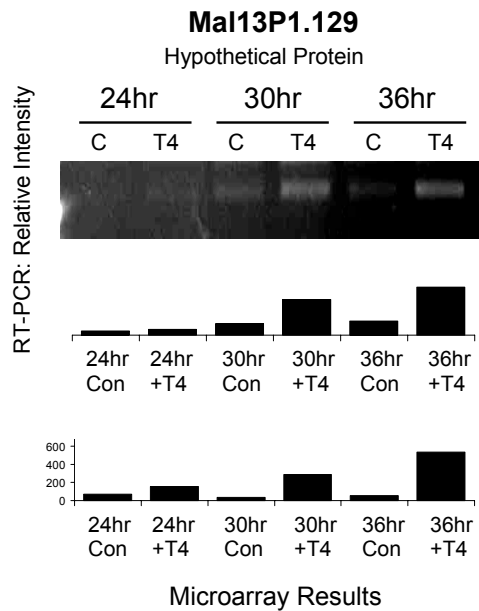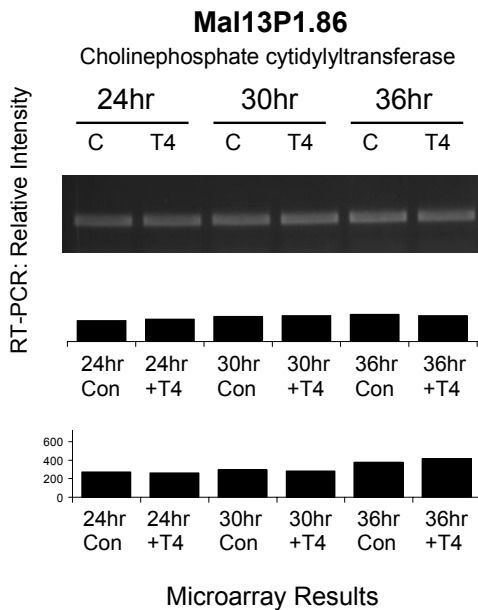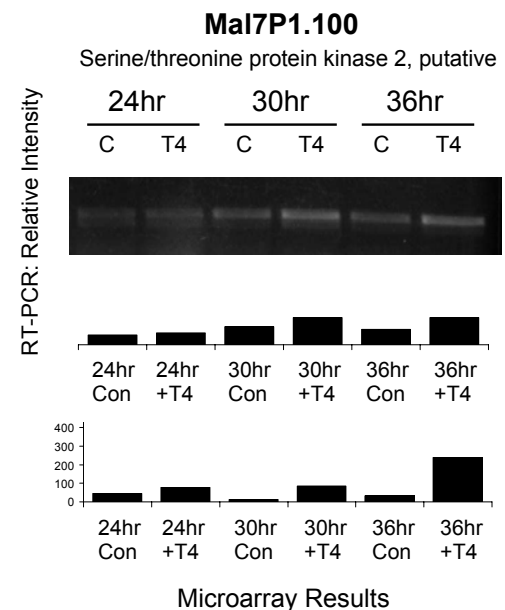

Supplement: Additional file 1 — Additional Figure 1: RT-PCR validation – RT-PCR experiments were performed to validate the microarray results. Plasmodium RNA was extracted at 24, 30, and 36 hours after 40 nM T4 treatment of synchronized cultures. Untreated RNA was extracted in tandem as a control. cDNA was created from the RNA extractions and quantified using spectrophotometry. 15 ng of cDNA were loaded into each RT-PCR reaction and was subjected to 20–35 cycles of amplification. Semi-quantifications of RT-PCR were done using NIH's ImageJ and illustrated by a column graph. Another column graph depicting the microarray results for the respective genes is shown for comparison. Pf14_0708, Mal13P1.129 and Mal7P1.100 were selected as genes expressed in gametocytes and showing a significant induction when incubated with T4); PF11_0509 and Mal7P1.6 showed an arrest of the cell cycle progression. Mal13P1.86, a gene involved in the parasite lipidic pathway is expressed and does not show any particular transcriptional change when incubated with the drug. [file 1471-2164-9-513-S1.pdf]
